# Rapid desensitization in delayed hypersensitivity reactions to chemotherapy and monoclonal antibodies

**DOI:** 10.1186/2045-7022-4-S3-P59

**Published:** 2014-07-18

**Authors:** Arantza Vega, Ana M Alonso, Belen Mateo, Juan M Beitia

**Affiliations:** 1 Gerencia de Atención Integrada de Guadalajara, Allergy Service, Spain

## Background

Drug desensitization is the induction of temporary clinical unresponsiveness to drug antigens to which patients have presented severe hypersensitivity reactions (HSR). Rapid desensitization in patients suffering immediate hypersensitivity reactions with chemotherapeutic agents and monoclonal antibodies have been widely described and have shown to be successful. Non-immediate hypersensitivity reactions with other drugs have usually required desensitization with several days' protocols to achieve total doses.

## Method

Forty three desensitization procedures were performed in 6 patients with a 12-13 step, 6-hour protocol. All patients had developed a delayed maculopapular rash with the use of chemotherapeutic and/or biological agents. Four patients were pretreated with corticosteroids, paracetamol and antihistamines before each desensitization procedure.

## Results

All the 43 desensitizations undertaken were successfully completed (Temozolamide 25, Bendamustine 4, Rituximab 4, Infliximab 5 and Gemcitabina 5). We observed HSR during 11 (25%) of desensitizations, including 5 inmediate exantema, 3 delayed local macular exantema and 3 delayed maculopapular lesions. Four patients were treated with corticosteroids and anti-histamines after the desensitization protocol to avoid more delayed HSR.

## Conclusions

Rapid desensitization protocols are safe and effective in getting over delayed HSR to chemotherapeutic and monoclonal antibodies and allow patients with severe diseases to continue their treatment.

